# P-82. IV to Oral: Outcomes in the Treatment of Staphylococcus aureus Osteomyelitis

**DOI:** 10.1093/ofid/ofaf695.311

**Published:** 2026-01-11

**Authors:** Alison M Beieler, Kristine F Lan, Shireesha Dhanireddy, Chloe Bryson-Cahn, Audrey Li, Jeannie D Chan, Whitney Hartlage, Preston Kramer, H Nina Kim, Jason Simmons

**Affiliations:** Harborview Medical Center, Seattle, WA; University of Washington, Seattle, Washington; University of Washington, Seattle, Washington; Harborview Medical Center, Seattle, WA; University of Washington, Seattle, Washington; University of Washington, Seattle, Washington; Harborview Medical Center, UW Medicine, Seattle, WA, Seattle, WA; University of Washington, Seattle, Washington; University of Washington, Seattle, Washington; University of Washington, Seattle, Washington

## Abstract

**Background:**

Outcomes for patients receiving oral antibiotics for *Staphylococcus aureus* (SA) osteomyelitis, including methicillin-resistant SA (MRSA), are poorly characterized. We evaluated outcomes in a high-risk population treated with oral therapy at a public safety-net hospital.

**Table 1.**
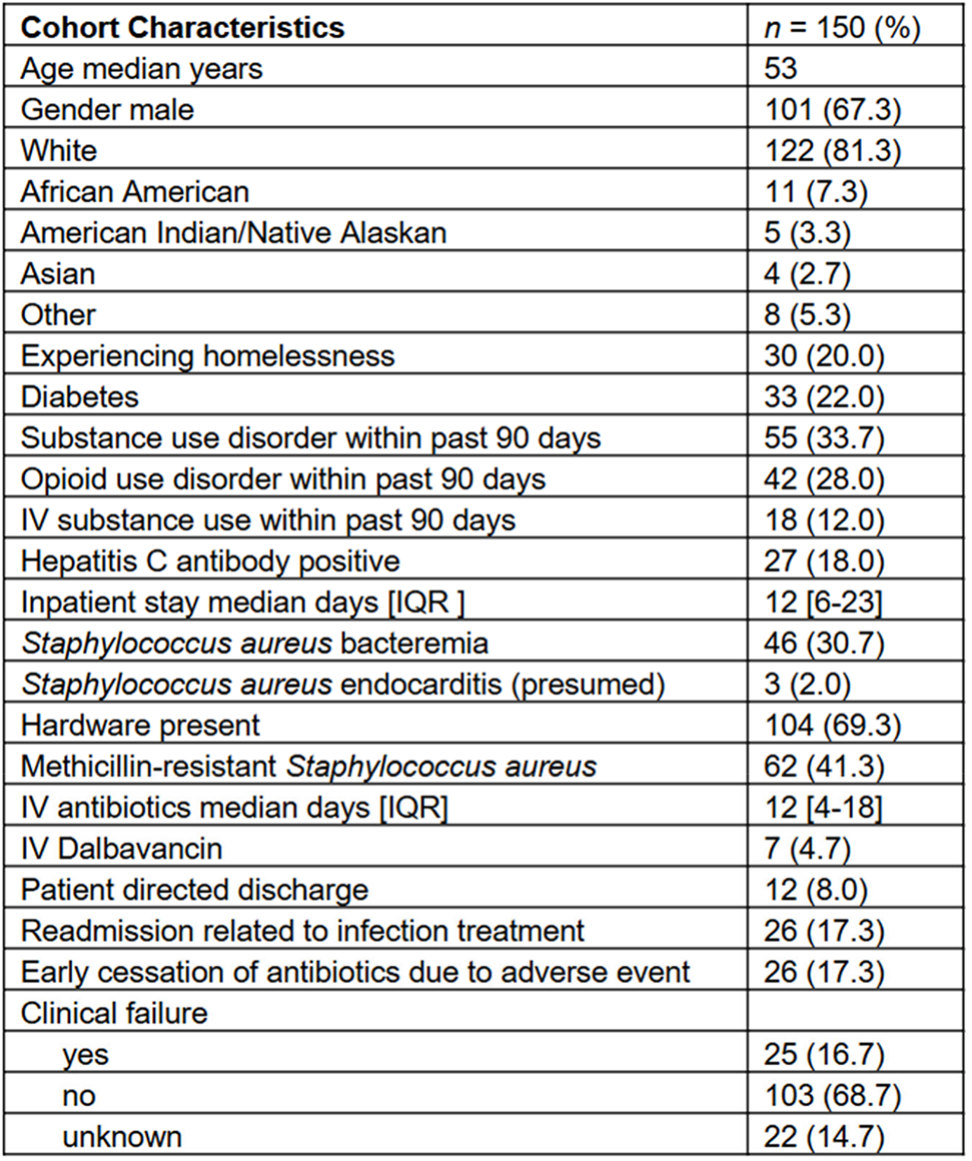
Cohort Characteristics

**Table 2.**
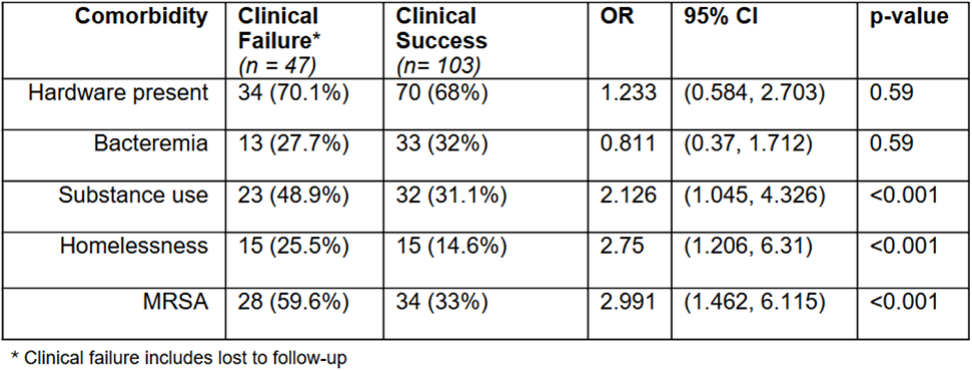
Association between clinical failure and patient comorbidities within osteomyelitis cohort

**Methods:**

All hospitalized adults with SA isolated from microbiologic specimens were identified via electronic query from 6/30/2021 to 6/30/2024 at Harborview Medical Center. Demographic data and event dates were extracted and imported into REDCap. Diagnosis of osteomyelitis (1st episode per anatomic location) was confirmed by chart review, as were clinical features (bacteremia, hardware, antibiotic duration), comorbidities and outcomes (loss to follow-up, reason for readmission). Clinical failure was defined as lack of antibiotic completion, relapse, readmission due to infection within 90 days of discharge, or loss to follow-up. Patients who received only intravenous (IV) antibiotics or those who underwent definitive amputation were excluded. Association between covariates was assessed using chi-square tests or univariate logistic regression.

**Results:**

We identified 150 individuals with SA osteomyelitis including bacteremia (31%), MRSA (41%), and hardware (69%) infections, all partially treated with oral therapy (Table 1); 146 underwent aspiration or debridement. Nearly all (97%) received IV therapy initially (median duration 12, IQR 4-18 days). Seven received dalbavancin. Adjunctive rifampin was used in 90 (60%) individuals. Relapse or readmission due to treatment complication occurred in 25 (17%) patients and 22 were lost to follow up. Duration of IV therapy > 2 weeks was associated with presence of hardware (2.5 OR; CI 1.1-5.7) and bacteremia (4.2 OR; CI 2.0-8.8), and neither of these were associated with clinical failure. Substance use disorder (OR 2.12; CI 1.0-4.3), homelessness (OR 2.75; CI 1.2-6.3), and MRSA (OR 2.99; CI 1.5-6.1) were associated with clinical failure (Table 2).

**Conclusion:**

Despite a high-risk population, the majority of patients were successfully treated for SA osteomyelitis. These findings highlight the need for targeted interventions addressing housing instability and substance use to improve osteomyelitis treatment outcomes.

**Disclosures:**

All Authors: No reported disclosures

